# Invariance to frequency and time dilation along the ascending ferret auditory system

**DOI:** 10.1186/1471-2202-16-S1-P51

**Published:** 2015-12-18

**Authors:** Alexander G Dimitrov, Jean F Lienard, Zachary Schwartz, Stephen V David

**Affiliations:** 1 Department of Mathematics, Washington State University Vancouver, Vancouver, WA 98686, USA; 2 Department of Otolaryngology, Oregon Health Sciences University, Portland, OR 97239, USA

## 

The sense of hearing requires a balance between competing processes of perceiving and ignoring. Behavioral meaning depends on the combined values of some sound features but remains invariant to others. The invariance of perception to physical transformations of sound can be attributed in some cases to local, hard-wired circuits in peripheral brain areas. However, at a higher level this process is dynamic and continuously adapting to new contexts throughout life. Thus the rules defining invariant features can change.

In this project, we test the idea that high-level, coherent auditory processing is achieved through hierarchical bottom-up combinations of neural elements that are only locally invariant. The main questions we address in the context of an auditory system are: 1. What kinds of changes in sound do **not **affect initial stages of auditory processing? 2. How does the brain manipulate these small effects to achieve a coherent percept of sounds?

Local probabilistic invariances, defined by the distribution of transformations that can be applied to a sensory stimulus without affecting the corresponding neural response [1,2], are largely unstudied in auditory cortex. We assess these invariances at two stages of the auditory hierarchy using single neuron recordings from the primary auditory cortex (A1) and the secondary auditory cortex (PEG) of awake, passively listening ferrets [3,4].

Our results show that stimulus invariance to frequency and time dilations are present at every tested stage and increase along the hierarchical auditory processing. At least in the early stages, parametric models having invariance properties by design are well-suited to describing biological functions. We were further able to characterize meaningful relationships among receptive field shapes. Preliminary observations indicate that joint time/frequency receptive fields are oriented toward central frequencies; receptive field widths are proportional to the best frequency; and late-onset neurons are also exhibiting the most sustained activity.

**Figure 1:**
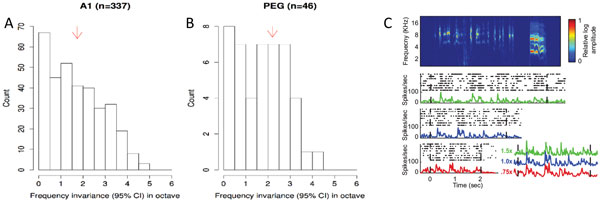
**95% confidence interval in frequency for the highest responses in A1 (**A**) and PEG (**B**) as tested with narrowband noise stimuli**. The higher values found in PEG compared to A1 demonstrate higher invariance to frequency shifts in PEG than in A1for both stimuli. (**C**) A ferret vocalization (top) and three responses to different time dilations (bottom). The inset shows the aligned firing rates, indicating similarities and differences in the responses.
